# Assessment of Alveolar Bone and Periodontal Status in Peritoneal Dialysis Patients

**DOI:** 10.3389/fphys.2021.759056

**Published:** 2021-12-13

**Authors:** Kristine Sun, Hui Shen, Yingli Liu, Hai Deng, Huiwen Chen, Zhongchen Song

**Affiliations:** ^1^Department of Periodontology, Shanghai Ninth People’s Hospital, Shanghai Jiao Tong University School of Medicine, College of Stomatology, Shanghai Jiao Tong University, National Center for Stomatology, National Clinical Research Center for Oral Diseases, Shanghai Key Laboratory of Stomatology, Shanghai, China; ^2^Department of Nephrology, Shanghai Ninth People’s Hospital, Shanghai Jiao Tong University School of Medicine, Shanghai, China

**Keywords:** chronic kidney disease, peritoneal dialysis, periodontal status, alveolar bone, cone-beam computed tomography (CBCT)

## Abstract

Chronic kidney disease (CKD) affects 8–13% of the global population and has become one of the largest burdens on healthcare systems around the world. Peritoneal dialysis is one of the ultimate treatments for patients with severe CKD. Recently, increasing severe periodontal problems have been found in CKD patients. Periodontitis has been identified as a new variable risk factor for CKD. The aim of this study was to investigate the periodontal status and severity of alveolar bone loss in CKD patients with peritoneal dialysis (PD). One hundred and six patients undergoing PD (PD group) and 97 systemically healthy periodontitis patients (control group) were enrolled. The differences in the dimensions of the alveolar bone between two groups were compared, and the distribution of alveolar bone defects was analyzed by cone-beam computed tomography (CBCT). Gingival index (GI), plaque index (PLI), periodontal probing depth (PPD), and attachment loss (AL) were recorded. The levels of inflammatory factors in gingival crevicular fluid were assessed by ELISA. Compared to control group, there was a higher degree of alveolar bone loss in maxillary premolars, maxillary 2nd molar and mandibular 1st molar in patients with PD (*p* < 0.05). A comparison of bone loss in different sites revealed that the area with the highest degree of bone loss were on the mesial-buccal, mid-buccal, distal-buccal, and mesial-lingual site in PD patients. The expression levels of inflammatory factors were higher in PD group (*p* < 0.01). In conclusion, PD patients presented more severe periodontal and inflammatory status than systemically healthy periodontitis patients. The loss of the alveolar bone differed between the two groups. Different sites and teeth exhibited a diverse degree of bone loss. This study highlights that clinicians should pay close attention to periodontal status of peritoneal dialysis patients and provides a new thinking to improve healthcare for CKD.

## Introduction

Periodontitis is a chronic inflammatory and destructive disease characterized by periodontal tissue damage. Recent global burden of disease studies have shown that severe periodontitis is the sixth-most prevalent disease worldwide (Tonetti et al., 2017; Hickey et al., 2020). Individuals with periodontitis are at risk of multiple tooth loss and masticatory dysfunction, thereby impairing their nutrition and lowering their quality of life (Tonetti et al., 2017). Since the 1990s, the link between periodontal health and systemic conditions has been increasingly noted, leading to the development of “Periodontal Medicine.” Periodontitis is thought to affect several common systemic conditions such as chronic kidney disease (CKD), cardiovascular disease (CVD), and diabetes mellitus. Periodontal medicine establishes a two-way relationship between periodontal disease and overall health (Monsarrat et al., 2016). Recently, the relationship between periodontitis and CKD has become a research hotspot.

Chronic kidney disease is characterized by either a glomerular filtration rate (GFR) of < 60 mL/min/1.73 m^2^ or detection of markers of kidney damage for at least 3 months. It has become a worldwide public health concern with its high prevalence, high treatment cost and severe complications (Tasdemir et al., 2018). The development of glomerular injury and failure of the glomerular filtration barrier can trigger a cascade of events that can finally lead to kidney failure (KF). The latter is characterized by a GFR of < 15 mL/min/1.73 m^2^ or treatment by dialysis (Levey et al., 2020).

Kidney replacement therapy is one of the main life-saving medical procedures for KF patients. Peritoneal dialysis (PD) and hemodialysis (HD) are currently two of the universally performed procedures as part of kidney replacement therapy (Miyata et al., 2019). Periodontitis is very common in patients with CKD, especially in population undergoing dialysis (Kshirsagar and Grubbs, 2015), which can increase the risk of CKD (Kapellas et al., 2019). Ricardo et al. (2015) found that periodontitis may be a non-traditional risk factor of CKD, which will accelerate the progress of CKD.

With the gradual decline of GFR, metabolic disorders of minerals and bone are common in CKD patients (Aleksova et al., 2018). Chronic kidney disease-mineral and bone disorder (CKD-MBD) is one of the most common complications in patients with CKD (Levey et al., 2020). A study involving 102 HD patients and 204 systemic healthy subjects found that patients on HD had more attachment loss than healthy people, and cone-beam computed tomography (CBCT) results showed that HD patients had more severe alveolar bone loss (Zhao et al., 2014). Gupta et al. (2018) also reported more severe periodontal problems and periodontal bone loss in hemodialysis patients than in the general healthy population. However, few studies have been conducted on alveolar bone and the periodontal status of patients undergoing PD.

Therefore, the aim of this study was to evaluate the volumetric change in alveolar bone in PD patients and analyze the distribution of bone loss. In addition, a comparison between PD patients and periodontitis patients for periodontal status was also carried out.

## Materials and Methods

The study design was approved by the Institutional Ethics Committee of the Shanghai Ninth People’s Hospital Affiliated to Shanghai Jiao Tong University School of Medicine prior to the implementation of the study (No.2018-120-T98). Written informed consent was obtained from all participants before enrollment.

### Study Population

**FIGURE 1:**
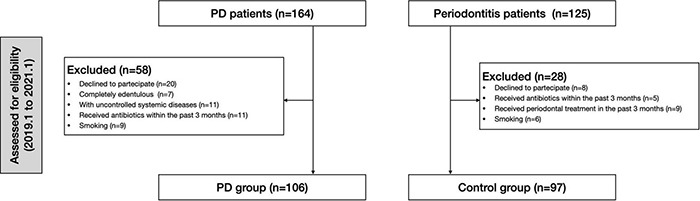
Flow chart of the study. PD, peritoneal dialysis.

This is an observational cross-sectional study of 106 PD patients and 97 periodontitis patients without any underlying systemic disease enrolled between January 2019 and February 2021 from Shanghai Ninth People’s Hospital attached to Shanghai Jiao Tong University School of Medicine (Figure 1).

The inclusion criteria of PD patients (PD group) were as follows: (1) aged between 18 and 80 years; (2) ≥ 10 natural teeth present in the oral cavity (excluding the third molar); and (3) estimated GFR (eGFR) < 10 mL/min/1.73 m^2^ (based on CKD-EPI creatinine equation) and receipt of regular stable PD for more than 3 months. PD patients were subsequently divided into three subgroups based on PD vintage.

The controls were recruited consecutively from the Department of Periodontology, Shanghai Jiao Tong University School and matched with the PD patients based on sex and age (within 5 years). The inclusion criteria of systemically healthy periodontitis patients (control group) were: (1) aged between 18 and 80 years; (2) no underlying systemic disease; (3) diagnosis of generalized stage III and IV (grades A to C) periodontitis (according to the 2018 periodontal disease classification); (4) ≥ 10 natural teeth present in the oral cavity (excluding the third molar).

The exclusion criteria were as follows: (1) completely edentulous patients; (2) those with liver cirrhosis or cancer; (3) those with other malignant tumors; (4) those with a history of any periodontal treatment in the past 3 months; (5) those who received antibiotics and/or immunosuppressive therapy within the past 3 months; (6) pregnant women; (7) those with uncontrolled systemic diseases like diabetes; (8) unable or unwillingness to sign the informed consent form; and (9) smokers.

CBCT images were excluded according to the following criteria: (1) unclear visibility of anatomical landmarks, cemento-enamel junction (CEJ), and alveolar bone crest (ABC); (2) visibility of the CEJ was compromised by the presence of restorations, prostheses, and other artifacts; and (3) imaging was limited by too many metal artifacts.

### Periodontal Clinical Examination

Both the included PD patients and periodontitis patients underwent full-mouth clinical periodontal examination by a trained and calibrated periodontist (CHW). The examination comprised measurements of the gingival index (GI), periodontal probing depth (PPD), plaque index (PLI), and clinical attachment loss (CAL). The clinical evaluation was performed by UNC-15 periodontal probe (Hu-Friedy, Chicago, United States). Six sites (mesio-buccal, mid-buccal, distal-buccal, mesio-lingual, mid-lingual, and distal-lingual) per tooth were measured.

### Periodontal Bone Loss Assessments

**FIGURE 2:**
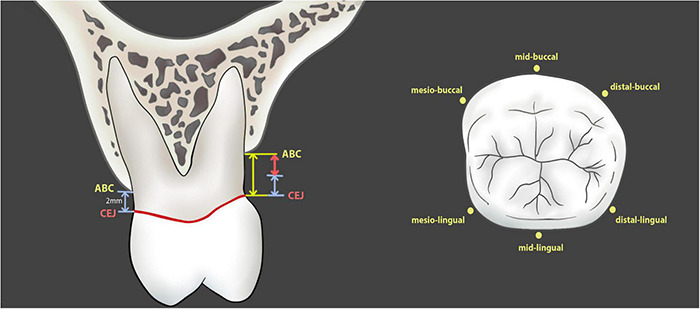
Measurement of alveolar bone loss in patients and in CBCT images. The numerical value of alveolar bone loss is the distance from alveolar bone crest to cementoenamel junction minus 2 mm (red line with double arrow).

**FIGURE 3:**
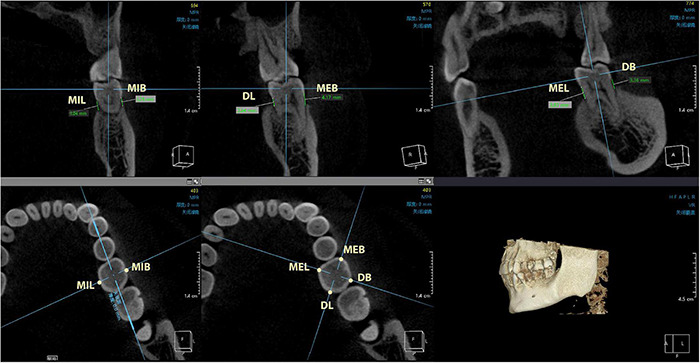
The measurement points include mesio-buccal, mid-buccal, distal-buccal, mesio-lingual, mid-lingual, and distal-lingual sites. CEJ, cementoenamel junction; ABC, alveolar bone crest; MEB, mesial-buccal; MIB, mid-buccal; DB, distal-buccal; MEL, mesial-lingual; MIL, mid-lingual; DL, distal-lingual.

Twenty subjects were selected from each group (PD group or control group) to undergo cone beam computed tomography (CBCT) scan (KaVo 3D eXam i-CAT, Germany). The digital imaging and communication in medicine (DICOM) files were exported to the Invivo5 software (Anatomage, San Jose, CA, United States) for subsequent measurements. The distance from the CEJ to the ABC was measured at 6 sites (mesio-buccal, mid-buccal, distal-buccal, mesio-lingual, mid-lingual, and distal-lingual) (Figures 2, 3), and the average of the recordings was used as a measure of the bone loss for one tooth in millimeters. Alveolar bone loss was established when the distance between the CEJ and the alveolar bone crest was greater than 2 mm.

### Gingival Crevicular Fluid Sampling and Analysis

After periodontal clinical examination, GCF samples of subjects were obtained from the Ramfjord teeth (#16, #21, #24, #36, #41, and #44) and the teeth with PPD ≥ 5 mm. Before GCF collection, the tooth was air-dried, the supragingival plaque was gently removed and clean cotton rolls were used to isolate the tooth surfaces. A Periopaper^®^ (Oraflow Inc., NY, United States) filter strip was inserted gently and kept in the gingival crevicular for 30 s. A total of 20 strips were collected from each subject and the weight (milligram, mg) was calculated. Strips contaminated with blood and/or saliva were discarded, and the correct specimens were placed in sterile 1.5-mL EP tubes and stored at -80°C until further use.

Prior to the inflammatory factor analysis, GCF samples were dissolved in 1ml-PBS solution (pH 7.2). After centrifuging the tubes (4°C, 1,500*g, 15 min), the supernatant was collected and immediately assessed for interleukin (IL)-1β, IL-8, IL-6, tumor necrosis factor (TNF)-α, and high-sensitivity C-reactive protein (hs-CRP) by enzyme-linked immunosorbent assay kits (IL-6, TNF-α, and hs-CRP: Signalway Antibody, United States; IL-8: Absin Bioscience Inc, China; IL-1β: NeoBioscience, China), according to the manufacturer’s instructions. The amount of IL-1β, IL-8, IL-6, and TNF-α were expressed as picograms. The amount of hs-CRP was expressed as nanograms. Cytokine concentrations (pg/mg) and hs-CRP concentrations (ng/mg) were calculated from the weight of GCF: total cytokine (pg)/total weight GCF (mg) or total hs-CRP (ng)/ total weight GCF (mg) (Dutzan et al., 2009).

### Statistical Analysis

All data was analyzed using SPSS software (version 20; IBM Corporation, Armonk, NY, United States) for Microsoft Windows. The normality was evaluated by the Shapiro-Wilk test. The normally distributed variables were described using the mean and standard deviation (SD), while the median and interquartile range (IQR) was used to describe non-normally distributed data. Chi-squared tests were used for categorical variables. Statistical significance was determined using an independent samples *t*-tests or analysis of variance (ANOVA) if the results were normally distributed. Wilcoxon rank-sum test or Kruskal-Wallis test were used if the results were non-normally distributed. Bonferroni correction was applied to the multiple comparisons. Correlation analysis was performed by Spearman’s or Pearson’s correlation method for abnormally or normally distributed data. The *p*-values<0.05 was considered to indicate statistical significance. Patients’ identities were hidden during data analysis. This study was performed according to the STROBE checklist.

## Results

### Baseline Characteristics and Clinical Parameters

A total of 106 patients (61 males and 45 females, mean age: 58.7 ± 14.3 years) and 97 patients (52 male and 45 female, mean age: 56.2 ± 10.1 years) were included in the PD and control groups, respectively. As shown in Table 1, there was no significant difference between age and sex. The primary cause of KF in PD patients was unknown (76.2%), 10.4% had diabetic kidney disease (DKD), and 3.8% had chronic glomerulonephritis and immunoglobulin A nephropathy (IgAN). Others included polycystic kidney, minimal glomerulonephritis, obstructive nephropathy, and systemic lupus erythematosus. The PD vintage was distributed as follows: 64 (60.38%) were 25–60 months, 34 (32.07%) were 3–24 months, and 8 (7.54%) were > 60 months (Table 1).

**TABLE 1:** Patients’ basic characteristic.

	Control group (*n* = 97)	PD group (*n* = 106)	*p*-value
Age (years)	56.2 ± 10.1	58.7 ± 14.3	0.754
M/F (*n*)	52/45	61/45	0.857

**PD group**			

**Primary etiology**			
Diabetes	11 (10.4%)		
Chronic glomerulonephritis	4 (3.8%)		
Immunoglobulin A nephropathy	4 (3.8%)		
Polycystic kidney	1 (0.9%)		
Minimal glomerulonephritis	1 (0.9%)		
Obstructive nephropathy	1 (0.9%)		
Systemic lupus erythematosus	1 (0.9%)		
Nephrotic syndrome	1 (0.9%)		
Mesangial proliferative glomerulonephritis	1 (0.9%)		
Unknown	80 (76.2%)		
**PD vintage**			
3∼24 months	34 (32.1%)		
25∼60 months	64 (60.4%)		
> 60 months	8 (7.5%)		
**Comorbidities and relevant medications**			
Hypertension (Calcium ion antagonist; diuretic; angiotensin II receptor blocker; etc.)	75 (70.7%)		
Diabetes (Melbine; insulin; acarbose; etc.)	24 (22.6%)		
Renal Anemia (Hemopoietin)	18 (17.0%)		
Abnormal bone metabolism status (Caltrate; Calcitriol; etc.)	18 (17.0%)		
Hyperphosphatemia (Calcium Acetate Tablets; lanthanum carbonate)	26 (24.5%)		
Hyperlipidemia (Statins)	3 (2.8%)		

*PD, peritoneal dialysis; M/F, Male/Female.*

Table 2 shows the biochemical parameters of the PD group at baseline. The median ALP, intact PTH, and hs-CRP were 84.00 U/L, 230.75 pg/mL, and 4.94 mg/L, respectively. The mean values of serum calcium (Ca), phosphorus (P), and 25-OH Vitamin D were 2.22 mmol/L, 1.78 mmol/L, and 9.85 ng/mL, respectively.

**TABLE 2:** Laboratory data of PD patients at baseline.

	PD Group (*n* = 106)	Normal value
**Serum biochemical markers**		
Ca (mmol/L)	2.22 ± 0.25 (95%CI: 2.17–2.27)	2.08–2.65
P (mmol/L)	1.78 ± 0.55 (95%CI: 1.67–1.89)	0.78–1.65
ALP (U/L)	84.00 (72.00–119.50)	46.00–116.00
Intact PTH (pg/mL)	230.75 (141.67–415.20)	12.00–88.00
25-OH Vitamin D (ng/mL)	9.85 ± 0.47 (95%CI: 8.90–10.79)	30.00–100.00
hs-CRP (mg/L)	4.94 (0.85–17.05)	0.00–10.00

*PD, peritoneal dialysis; Ca, calcium; P, phosphate; ALP, alkaline phosphatase; PTH, parathyroid hormone; hs-CRP, high-sensitivity C-reactive protein.*

### Periodontal Status

Table 3 depicts the periodontal clinical parameters with statistical comparisons for different PD vintage of PD groups and the control group. No statistical differences were observed among the four groups for CAL (*p* = 0.109). The PPD, GI, and PLI were significantly different (*p* < 0.0001, *p* = 0.001, *p* = 0.004) among four groups.

**TABLE 3:** Periodontal clinical parameters in patients with periodontitis (Control group) and peritoneal dialysis (PD group) (Mean ± SD).

	Control group (*n* = 97)	PD group (*n* = 106)			*F*	*p* value
		PD vintage: 3∼24 months	PD vintage: 25∼60 months	PD vintage: >60 months		
PPD (mm)	3.22 ± 0.81	3.66 ± 0.76	3.72 ± 0.96^a^	4.53 ± 0.68^a^,^b^,^c^	24.268	<0.0001
CAL (mm)	3.68 ± 0.71	4.02 ± 1.19	4.01 ± 1.09	4.04 ± 1.75	2.049	0.109
GI	1.73 ± 0.24	1.82 ± 0.21	1.85 ± 0.20^a^	1.91 ± 0.26^a^	5.827	0.001
PLI	1.72 ± 0.37	1.84 ± 0.44	1.85 ± 0.41	2.23 ± 0.41^a^	4.523	0.004

*PD, peritoneal dialysis; PPD, periodontal probing depth; CAL, clinical attachment loss; GI, gingival index; and PLI, plaque index.*

*^a^Significant change from Control group.*

*^b^Significant change from PD Group (PD vintage: 3∼24 months).*

*^c^Significant change from PD Group (PD vintage: 25∼60 months).*

Regarding the PPD, PD patients with PD vintage > 60 months exhibited an average of 4.53 ± 0.68 mm, significantly more than other PD patients with shorter vintage and control group. For GI, the control group exhibited an average of 1.73 ± 0.24, while patients with PD vintage > 60 months, 25–60 months, and 3–24 months showed a mean of 1.91 ± 0.26, 1.85 ± 0.20, and 1.82 ± 0.21, respectively; the difference was statistically significant between the control group and patients with PD vintage > 24 months (*p* < 0.01). There were significant differences in PLI values between the PD group with PD vintage > 60 months and the control group (*p* < 0.001).

### Inflammatory Factors in the GCF of Peritoneal Dialysis Patients

**FIGURE 4:**
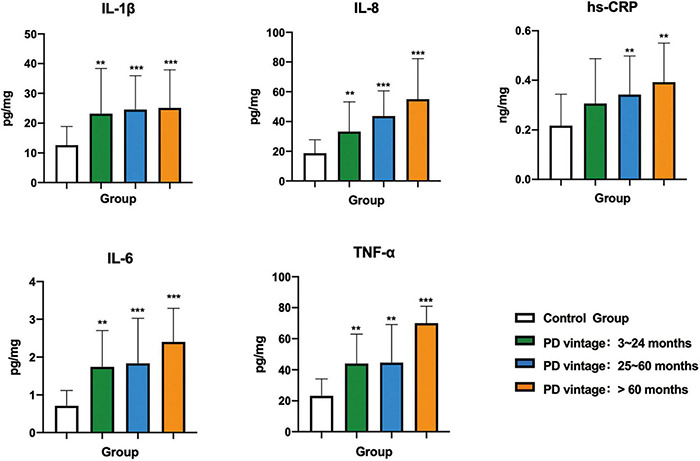
Comparison of inflammatory factors in gingival crevicular fluid among control group and PD groups with different vintage. Significant different from control group: *p < 0.05; **p < 0.01; ***p < 0.001.

As shown in Figure 4 and Table 4, the levels of IL-1β, IL-6, IL-8, TNF-α, and hs-CRP in the control group were all statistically significantly lower than each PD subgroups (*p* < 0.01). There was no significant difference among the three subgroups.

**TABLE 4:** Inflammatory factors in patients with periodontitis (Control group) and peritoneal dialysis (PD group) (Mean ± SD).

	Control group (*n* = 30)	PD group (*n* = 30)			*F*	*p* value
		PD vintage: 3∼24 months	PD vintage: 25∼60 months	PD vintage: >60 months		
IL-1β (pg/mg)	13.38 ± 6.98	23.17 ± 15.17^a^	24.54 ± 11.24^a^	25.16 ± 12.71^a^	5.273	0.003
hs-CRP (ng/mg)	0.22 ± 0.13	0.31 ± 0.18	0.34 ± 0.15^a^	0.39 ± 0.16^a^	7.335	0.012
IL-6 (pg/mg)	0.67 ± 0.36	1.74 ± 0.96^a^	1.83 ± 1.19^a^	2.40 ± 1.00^a^	10.246	<0.0001
TNF-α (pg/mg)	23.25 ± 10.85	43.96 ± 18.91^a^	44.66 ± 24.42^a^	70.07 ± 10.80^a^	8.686	<0.0001
IL-8 (pg/mg)	18.81 ± 8.96	33.29 ± 19.94^a^	43.73 ± 16.86^a^	55.05 ± 27.15^a^	13.780	<0.0001

*PD, peritoneal dialysis; PPD, periodontal probing depth; CAL, clinical attachment loss; GI, gingival index; PLI, plaque index.*

*^a^Significant change from Control Group.*

### Alveolar Bone Resorption

PD patients had severe alveolar bone loss. A comparison of bone loss between different jaws revealed a higher degree of alveolar bone loss in all teeth except the maxillary first molar and mandibular premolars (*p* < 0.05). For the mandibular lateral incisor, the bone loss in the PD group was significantly higher than other teeth, compared with the control group (*p* < 0.001) (Table 5).

**TABLE 5:** The mean alveolar bone loss at different teeth (mm; mean ± SD).

	Control group (*n* = 20)	PD group (*n* = 20)	*p*-value
Maxillary central incisor	3.58 ± 0.81	4.32 ± 0.56	0.016*
Maxillary lateral incisor	3.28 ± 0.32	3.22 ± 0.41	0.003**
Maxillary canine	2.89 ± 0.31	3.91 ± 0.55	0.041*
Maxillary 1st premolar	3.05 ± 0.65	3.71 ± 0.44	0.008**
Maxillary 2nd premolar	3.02 ± 0.45	3.59 ± 0.27	0.001**
Maxillary 1st molar	3.39 ± 0.28	3.71 ± 0.49	0.062
Maxillary 2nd molar	3.42 ± 0.29	3.77 ± 0.34	0.012*
Mandibular central incisor	3.32 ± 0.24	3.75 ± 0.34	0.002**
Mandibular lateral incisor	2.98 ± 0.35	3.54 ± 0.34	< 0.001***
Mandibular canine	3.02 ± 0.66	3.71 ± 0.44	0.001**
Mandibular 1st premolar	3.41 ± 0.28	3.18 ± 0.33	0.074
Mandibular 2nd premolar	3.51 ± 0.39	3.99 ± 1.04	0.146
Mandibular 1st molar	2.81 ± 0.87	3.41 ± 0.26*	0.032*
Mandibular 2nd molar	3.26 ± 0.28	3.51 ± 0.21*	0.028*

PD, peritoneal dialysis; * p < 0.05; ** p < 0.01; *** p < 0.001.

The distribution of alveolar bone loss at different sites of the same tooth was analyzed. The results revealed that bone resorptions in the mesio-buccal, mid-buccal, distal-buccal, mesio-lingual, and mid-lingual site of the PD group were statistical significantly greater than that of the control group (Table 6).

**TABLE 6:** Distribution of alveolar bone loss at different sites of the same tooth (mm; mean ± SD).

	Control group (*n* = 20)	PD group (*n* = 20)	*p*-value
Mesio-buccal	4.09 ± 1.51	3.53 ± 1.05	0.016*
Mid-buccal	3.83 ± 1.57	3.26 ± 1.36	0.031*
Distal-buccal	3.98 ± 1.74	3.37 ± 1.35	0.025*
Mesio-lingual	3.92 ± 1.26	2.91 ± 1.18	0.001**
Mid-lingual	3.47 ± 1.29	3.02 ± 1.05	0.032*
Distal-lingual	3.57 ± 1.37	3.26 ± 1.01	0.221

PD, peritoneal dialysis; *p < 0.05; **p < 0.01.

### Correlation Analyses

Robust relevance was observed between all inflammatory factors and periodontal indexes in the PD group, but not in the control group (Table 7). The correlation analysis did not identify a significant relationship between periodontal parameters and inflammatory factor levels, based on by very weak Spearman’s correlation coefficients in the control group. In addition, Spearman’s correlation found that PTH values had a relevant high degree of positive correlation with mean alveolar bone loss (*p* < 0.05). And there was an inverse correlation between 25-OH Vitamin D levels with mean alveolar bone loss (*p* < 0.05) (Table 8).

**TABLE 7:** Correlation analysis of inflammatory factors with periodontal parameters in different groups.

	PPD		CAL		GI		PLI	
	R^2^	*p*-value	R^2^	*p*-value	R^2^	*p*-value	R^2^	*p*-value
**PD group**								
IL-1β (pg/mg)	0.4789	<0.0001	0.2767	0.004	0.4290	<0.0001	0.2034	0.016
hs-CRP (ng/mg)	0.2735	0.005	0.3003	0.002	0.2841	0.003	0.0847	0.045
IL-6 (pg/mg)	0.2247	0.012	0.3795	<0.0001	0.5069	<0.0001	0.4970	<0.0001
TNF-α (pg/mg)	0.3136	0.008	0.2016	0.036	0.1954	0.040	0.3576	0.006
IL-8 (pg/mg)	0.4147	<0.0001	0.1884	0.021	0.3215	0.002	0.1911	0.002
**Control group**								
IL-1β (pg/mg)	0.0142	0.532	0.0003	0.928	0.0237	0.418	0.0018	0.824
hs-CRP (ng/mg)	0.0548	0.223	0.0119	0.572	0.0324	0.350	0.0449	0.269
IL-6 (pg/mg)	0.0408	0.285	0.0018	0.824	0.0104	0.690	0.0086	0.623
TNF-α (pg/mg)	0.1089	0.093	0.0630	0.207	0.1176	0.080	0.0552	0.237
IL-8 (pg/mg)	0.0202	0.454	0.0317	0.347	0.0096	0.605	0.0534	0.220

*PD, peritoneal dialysis; PPD, periodontal probing depth; CAL, clinical attachment loss; GI, gingival index; PLI, plaque index.*

**TABLE 8:** Correlation between PTH and 25-OH Vitamin D and mean alveolar bone loss in PD group.

	Mean alveolar bone loss (mm)	
	R	*p*-value
Intact PTH (pg/mL)	0.560	0.037
25-OH Vitamin D	−0.585	0.028

*PD, peritoneal dialysis; PTH, Parathormone.*

## Discussion

In the present study, assessment of full-mouth periodontal clinical parameters and alveolar bone assessments were carried out in both groups. The results of this study found that oral hygiene, degree of periodontitis, and alveolar bone resorption were more pronounced in PD patients than in the periodontitis population.

Chronic kidney disease is one of the most common chronic diseases with a worldwide prevalence estimated to be approximately 13.4% and projected to continue to rise annually (Hickey et al., 2020). Currently, an increasing number of KF patients are opting for PD given its simple equipment and operation, as it reduces the need to travel and can be performed at home. Some studies have demonstrated that patients with CKD have poor oral health and a high prevalence of periodontal disease (Kim et al., 2017; Gunupati et al., 2019; Lertpimonchai et al., 2019). A recent report suggested that 106 of 107 hemodialysis patients (99.1%) exhibit some symptoms of periodontitis, and another study also showed that only one of 103 hemodialysis patients evaluated had a healthy periodontium (Kim et al., 2017). Gupta et al. (2018) recruited 30 patients with kidney dialysis, 30 patients with pre-dialysis, and 30 healthy subjects to evaluate and compare the periodontal status of patients with systemic healthy individuals and patients who have KF before dialysis. The results showed that AL, PLI, and oral hygiene index-simplified (OHI-S) were more severe in patients undergoing kidney dialysis and patients with pre-dialysis than in subjects who were generally healthy. Tadakamadla et al. (2014) reported that the oral hygiene, gingival, and periodontal status decreased as the stage of CKD increased. Patients with KF showed poor oral hygiene and a higher prevalence of periodontal disease than healthy controls. In our study, we were surprised to find that all PD patients who met the inclusion criteria had periodontitis. Meanwhile, all periodontal parameters including the PPD, CAL, GI, and PI of PD patients were more severe than that in the control group, which indicated poor oral hygiene in PD patients.

Chronic kidney disease is often accompanied by disturbance in mineral metabolism. The main clinical manifestations are osteoporosis, osteitis fibrotic cystica, osteoarthritis, and pathological fracture. CKD is an independent risk factor for osteoporosis, and the incidence of fracture in patients with long-term dialysis is significantly higher than that in the general population (Evenepoel et al., 2017; Nakanishi et al., 2018; Li et al., 2019). Disturbance in mineral metabolism increases the risk of bone loss or bone fracture in patients with periodontitis (Kanjevac et al., 2018). Messier et al. (2012) obtained 129 orthopantomography (OPG) from dialysis patients, and the result showed that the extent of bone loss was higher among dialysis patients. Although OPG has the advantages of speed, low radiation, and low cost, it is only two-dimensional, thereby rendering it difficult to evaluate the width of the bone. Meanwhile, in traditional radiographs, the superimposition of anatomic structures and thickness of the roots may obscure many anatomical and pathological details. Comparatively, dental CBCT is highly sensitive and more comprehensive and significantly superior to the so-called conventional radiographs for analysis of alveolar bone loss. CBCT has been particularly widely used in clinical dentistry, as it could provide accurate periodontal bone loss. Therefore, CBCT was used to evaluate periodontal bone loss in this study. The results showed that the degree of alveolar bone loss was higher in all teeth than in the control group, except for the maxillary first molar and mandibular premolars (*p* < 0.05). Moreover, bone resorption was more pronounced in the PD group at the mesio-buccal, mid-buccal, distal-buccal, mesio-lingual, and mid-lingual sites than in the control group. However, owing to the complex root anatomy, it was not possible to accurately measure maxillary molar root bifurcation lesions in CBCT. Besides, in the present study, we also found that 25-OH Vitamin D showed negative association with the alveolar bone loss, and PTH level was positively associated with the alveolar bone loss. The majority of patients with CKDs are 25-OH Vitamin D deficient. Results of observational cross-sectional studies investigating the association between Vitamin D serum level and periodontitis indicate that, Vitamin D deficiency has been hypothesized to contribute to the pathogenesis of periodontitis in CKD. It perhaps owing to the immunomodulatory, anti-inflammatory, and antibacterial properties of 1,25(OH)_2_ D_3_/VDR signaling, a sufficient serum level of Vitamin D is necessary for the maintenance of periodontal health (Khammissa et al., 2018; Machado et al., 2020). PTH is a well-known stimulator of bone resorption. Thus, CKD-BMD is probably the most important risk factor/driver of alveolar bone loss.

Gingival crevicular fluid reflects the metabolic changes of periodontal support tissue. Collection and analysis of GCF have long been a popular approach to investigate localized inflammatory processes in periodontitis (Barros et al., 2016; Taylor and Preshaw, 2016). Dağ et al. (2010) detected that TNF-α and IL-8 levels in the GCF of HD patients were significantly higher than those of healthy people. The associated relationship between chronic systemic inflammation and CKD is measured according to hs-CRP levels. At present, there are limited studies on the expression level of inflammatory factors in the GCF of PD patients. The results of this study showed that the levels of IL-1β, IL-6, hs-CRP, IL-8, and TNF-α were higher in PD patients than those in the control group. Further, there were strong, positive correlations between clinical parameters and the levels of IL-1β, IL-6, hs-CRP, IL-8, and TNF-α in the GCF of PD patients. This showed that chronically elevated levels of these cytokines in PD patients may be linked to the more serious periodontal tissue destruction.

The main limitation of our study is that we did not evaluate alveolar bone loss by CBCT for each subject, because it is not a routine examination procedure for PD patients. Further, the limited sample size requires more work to verify and confirm the results. The second limitation of the study is that we did not evaluate the bone loss in furcation involvement because of the complex root anatomy. Last, we used only CBCT to evaluate bone loss; future studies should explore the ability of CBCT to predict bone density.

Globally, both CKD and periodontitis are associated with considerable healthcare-related burdens. However, periodontitis is always overlooked by the public as it is not a deadly disease, but it is in fact the most common oral disease worldwide. The high prevalence of periodontitis among PD patients indicates the need to increase awareness among clinicians on this condition. This study concluded that PD patients presented more severe periodontal status than systemically healthy periodontitis patients. The extension of the course of PD may aggravate periodontal damage. The loss of alveolar bone differed between the two groups. Different sites and teeth exhibited a diverse degree of bone loss. Clinicians should pay close attention to the periodontal status of PD patients. It is essential to improve the quality of life of PD patients and ensure early prevention of disease, along with offering effective treatment strategies for PD. Cone-beam CT has the potential to provide greater diagnostic information about alveolar bone loss than other imaging modalities. It is worthwhile to be generalized for further studies.

## Data Availability Statement

The raw data supporting the conclusions of this article will be made available by the authors, without undue reservation.

## Ethics Statement

The studies involving human participants were reviewed and approved by the Institutional Ethics Committee of the Shanghai Ninth People’s Hospital Affiliated to Shanghai Jiao Tong University School of Medicine (No. 2018-120-T98). The patients/participants provided their written informed consent to participate in this study.

## Author Contributions

KS executed the experiments, collected the data, and wrote the manuscript. HS analyzed all data and revised the manuscript. YL and HD enrolled participants and executed the experiments. HC designed the experiments and made the critical revision. ZS had made substantial contributions to conception and design, made the critical revision. All authors agreed to be accountable for the content of the work.

## Conflict of Interest

The authors declare that the research was conducted in the absence of any commercial or financial relationships that could be construed as a potential conflict of interest.

## Publisher’s Note

All claims expressed in this article are solely those of the authors and do not necessarily represent those of their affiliated organizations, or those of the publisher, the editors and the reviewers. Any product that may be evaluated in this article, or claim that may be made by its manufacturer, is not guaranteed or endorsed by the publisher.
